# Supplementary material for: Learning curve and surgical outcome of robotic assisted colorectal surgery with ERAS program
Source: Sci Rep. 2022 Nov 29;12:20566. doi: 10.1038/s41598-022-24665-w (PMC9709162; doi:10.1038/s41598-022-24665-w)
Supplement: Supplementary file 1 — Supplementary Information 1. [file 41598_2022_24665_MOESM1_ESM.zip › LSC Raw data-Ñ[▒K/101/O_ñw╝╨░O▒Kñσ.pdf]

正式摘要(電子簽章：000000202047347)

請選擇...reporter

臺中榮民總醫院

Operation Note

[redacted]

- 手術日期 : 2020/10/29
- 手術醫師 : VS 蔣鋒帆 , CR 陳映菁 , CR 林昌霖 , R 陳智翔
- 執行麻醉 : VS 王賜洲
- Anesthesia : GE
- Position : Lithotomy
- Aseptic agents : Soap , Betadine Alcohol , Betadine Aqua

Diagnosis

- Pre-operative diagnosis : Malignant tumor of T-colon,PROVED
- Post-operative diagnosis : Ditto.

Surgical Indication

- tumor removal

Operation

- 1. Laparoscopic assisted subtotal colectomy with side to side anastomosis, GIA 80/3.8*2, Re-inforcement with silk: nil, air leak test: not done
  2. Vessels ligation of ileo-colic, right colic, middle colic artery, left colic and IMA
  3. Cut margin: proximal 18cm(colon), 5cm(terminal ileum), distal 15cm
  4. Adhesion barrier film(+)

Finding

- 1. An ulcerative tumor, Bromann's type II, about 1cm x 2cm in size, at splenic flexure, which penetrated through muscular layer but didn’t invaded to serosa layer. cT2N0M0
  Another polypoid tumor, Bromann's type I, about 1*1cm in size, at hepatic flexure, tubular adenoa.
  2. No enlarged LNs was noted along the mesocolon or intermediate vessel
  3. There was no visible liver metastasis and no visible peritoneal seeding and carcinomatosis.
  4. 2 synchronous polyps, one is 0.5cm in size, and the other is 0.8cm in size at A-colon.

Procedure

- General endotracheal anesthesia was induced and then the patient was placed in modified Trendelenburg lithotomy position. Preoperative antibiotics were given. A Foley catheter and nasogastric tube were placed. The abdomen was prepped and draped in standard sterile fashion. A small vertical incision was made above the umbilicus and a 12mm trochar was inserted into the peritoneal cavity by open method. Peumoperitoneum was established with CO2 inflation. Laparoscopy was inserted and then another 12mm trochars were inserted over RLQ. Three 5mm trochars were inserted over RUQ, LUQ and LLQ of abdomen, respectively. The operative findings were mentioned as above. The small bowel was inspected and retracted to the right by grasper. The sigmoid colon was then held by grasper and the peritoneum overlying the mesentery was then scored with electrocautery. The inferior mesentery artery was identified, ligated near its origin from the aorta/below the left colic artery with endoclips, and transected, so was inferior mesentery vein. Tattoo for tumor location was identified at hepatic flexure and splenic flexuren. The right colon was mobilized by incising the peritoneal attachment from the cecum to the hepatic flexure. The greater omentum was then taken from the transverse colon up to the splenic flexure. The lateral peritoneal

- attachment of the left colon was then incised from the splenic flexure to the DS colon. The mesentery was scored and divided.A midline incision was made at para-umbilical region. wound retractor was inserted, and colon was extracted through this wound. ileum was ligated 5 cm proximal to IC valve with kocher clamp at specimen side and tied at proximal side, and then side-to-side anastomosis was applied by GIA 80/3.8*2 for terminal ileum and S-colon. Then anastomosis was re-enforced by 3-0 silk. Then the intestine was put back in to abdomen, and pneumoperitoneum was created again, bleeding was checked meticulously, and abdomen was irrigated carefully. Adhesion barrier was placed under paraumbilical wound. The wound was closed with interrupted sutures of No.1 Vicryl. The fascia of the two 12 mm trochar wound was closed with 1-0 Dexon. Subcutaneous tissues were closed with interrupted 3-0 Polysorb and the skin was closed with continue 4-0 Dexon. The patient tolerated the procedure well and was brought to the POR in stable condition.
- Estimated blood loss: minimum
- 器械數量: 正確
- 紗布數量: 正確

Image

- PAINT
  - colon & rectum

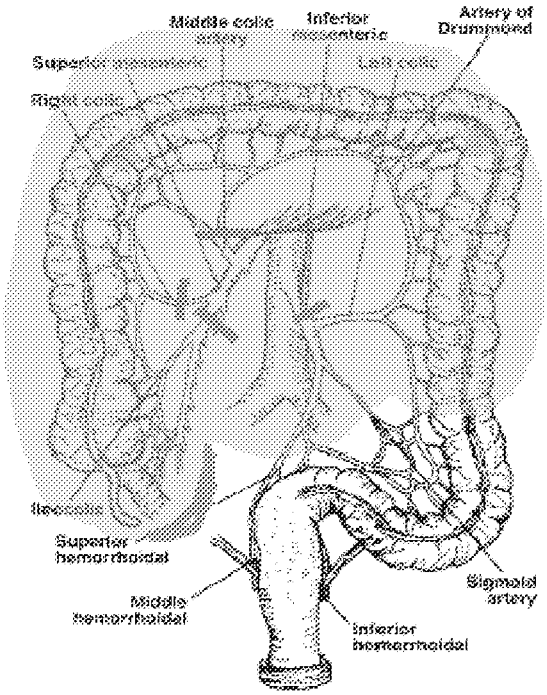

  - abdomen

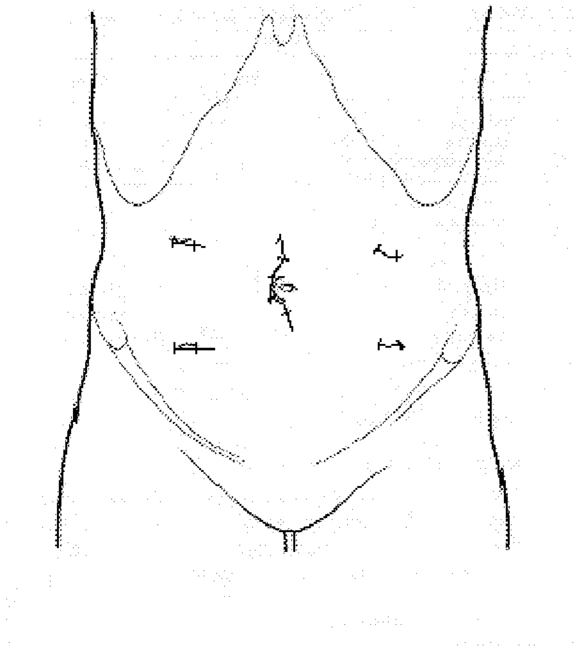

主任 2062F蔣鋒帆 / 醫師 2303K陳映菁

正式摘要**(**電子簽章：**000000202047347)**

請選擇...reporter

doctorRank:B
